# Supplementary material for: Device-embedded accelerometry complements neural signals for tracking parkinsonian motor states
Source: bioRxiv. 2026 Jul 9:2026.07.08.737286. Preprint. [Version 1] doi: 10.64898/2026.07.08.737286 (PMC13370933; doi:10.64898/2026.07.08.737286)
Supplement: Supplement 1 [file NIHPP2026.07.08.737286v1-supplement-1.pdf]

bioRxiv preprint doi: https://doi.org/10.64898/2026.07.08.737286; this version posted July 9, 2026. The copyright holder for this preprint (which was not certified by peer review) is the author/funder, who has granted bioRxiv a license to display the preprint in perpetuity. It is made available under a CC-BY 4.0 International license.

Appendix

Supplementary Table 1: Continuous DBS parameters used for each patient and hemisphere.

Subject	Hemisphere	Amplitudes (mA)	pulse width (us)	Frequency (Hz)	Stim Contact
RCS02	Left	2.4 - 2.6	60	130.2	c+2-
	Right	3.1 - 3.5	60	130.2	c+1-
RCS05	Left	1.8 - 3.0	60	130.2	c+1-
	Right	1.3 - 1.8	60	130.2	c+1-
RCS06	Left	1.2	60	158.7	c+2-
	Right	1.2	60	166.7	c+1-
RCS08	Left	2.7 - 4.7	60	130.2	c+2-
	Right	1.0 - 3.3	60	130.2	c+2-
RCS11	Left	2.3 - 3.5	60	130.2	c+2-
	Right	1.5 - 2	60	130.2	c+2-
RCS12	Left	1.7 - 3.2	60	130.2	c+2-
RCS15	Left	3.3 - 3.5	60	130.2 - 150.6	c+1-
	Right	3.8 - 4.4	70	130.2 - 150.6	c+1-
RCS17	Left	2.3 - 3	60	130.2	c+2-
	Right	1.6 - 2.5	60	129.9 - 130.2	c+2-
RCS20	Left	1.2 - 3.3	60	130.2	c+1-
	Right	1.2 - 2.1	60	130.2	c+1-

bioRxiv preprint doi: https://doi.org/10.64898/2026.07.08.737286; this version posted July 9, 2026. The copyright holder for this preprint (which was not certified by peer review) is the author/funder, who has granted bioRxiv a license to display the preprint in perpetuity. It is made available under a CC-BY 4.0 International license.

Supplementary Table 2: Feature Definitions

**Neural Features**	**Definition**
STN Low Beta Total Power	Mean total power of the low beta band (13-20 Hz) of the spectrum. Signals recorded in subthalamic nucleus
STN High Beta Total Power	Mean total power of the high beta band (20-35 Hz) of the spectrum. Signals recorded in subthalamic nucleus
STN Low Gamma Total Power	Mean total power of the low gamma band (40-70 Hz) of the spectrum. Signals recorded in subthalamic nucleus
STN High Gamma Total Power	Mean total power of the high gamma band (70-100 Hz) of the spectrum. Signals recorded in subthalamic nucleus
CTX Low Beta Total Power	Mean total power of the low beta band (13-20 Hz) of the spectrum. Signals recorded in motor cortex
CTX High Beta Total Power	Mean total power of the high beta band (20-35 Hz) of the spectrum. Signals recorded in motor cortex
CTX Low Gamma Total Power	Mean total power of the low gamma band (40-70 Hz) of the spectrum. Signals recorded in motor cortex
CTX High Gamma Total Power	Mean total power of the high gamma band (70-100 Hz) of the spectrum. Signals recorded in motor cortex
STN Low Beta Periodic Power	Mean periodic power of the low beta band (13-20 Hz) of the spectrum. Signals recorded in subthalamic nucleus
STN High Beta Periodic Power	Mean periodic power of the high beta band (20-35 Hz) of the spectrum. Signals recorded in subthalamic nucleus
STN Low Gamma Periodic Power	Mean periodic power of the low gamma band (40-70 Hz) of the spectrum. Signals recorded in subthalamic nucleus
STN High Gamma Periodic Power	Mean periodic power of the high gamma band (70-100 Hz) of the spectrum. Signals recorded in subthalamic nucleus
CTX Low Beta Periodic Power	Mean periodic power of the low beta band (13-20 Hz) of the spectrum. Signals recorded in motor cortex
CTX High Beta Periodic Power	Mean periodic power of the high beta band (20-35 Hz) of the spectrum. Signals recorded in motor cortex
CTX Low Gamma Periodic Power	Mean periodic power of the low gamma band (40-70 Hz) of the spectrum. Signals recorded in motor cortex
CTX High Gamma Periodic Power	Mean periodic power of the high gamma band (70-100 Hz) of the spectrum. Signals recorded in motor cortex
STN Aperiodic Offset	Mean aperiodic offset of spectrum. Signals recorded in subthalamic nucleus

bioRxiv preprint doi: https://doi.org/10.64898/2026.07.08.737286; this version posted July 9, 2026. The copyright holder for this preprint (which was not certified by peer review) is the author/funder, who has granted bioRxiv a license to display the preprint in perpetuity. It is made available under a CC-BY 4.0 International license.

STN Aperiodic Exponent	Mean aperiodic exponent of spectrum. Signals recorded in subthalamic nucleus
CTX Aperiodic Offset	Mean aperiodic offset of spectrum. Signals recorded in motor cortex
CTX Aperiodic Exponent	Mean aperiodic exponent of spectrum. Signals recorded in motor cortex
Low Beta Coherence	Mean coherence between subthalamic nucleus and motor cortex in the low beta band (13-20 Hz)
High Beta Coherence	Mean coherence between subthalamic nucleus and motor cortex in the high beta band (20-35 Hz)
Low Gamma Coherence	Mean coherence between subthalamic nucleus and motor cortex in the low gamma band (40-70 Hz)
High Gamma Coherence	Mean coherence between subthalamic nucleus and motor cortex in the high gamma band (70-100 Hz)

**Acceleration Features**	**Definition**
Max Acc	Maximum of the acceleration intensity within the selected window
Interquartile Acc	Difference between the 75th and 25th percentiles of the acceleration intensity within the selected window
90th Percentile Acc	Value of 90th percentile of the acceleration intensity within the selected window
Median Acc	Median of acceleration intensity within the selected window
Std Acc	Standard deviation of the acceleration intensity within the selected window
Var Acc	Variance of the acceleration intensity within the selected window
Mean Acc	Average of the acceleration intensity within the selected window
Coefficient of Variation	Ratio of standard deviation of acceleration intensity to mean intensity within the selected window, measuring the variability of signal
Min Acc	Minimum of the acceleration intensity within the selected window
Acc Entropy	Entropy of the acceleration intensity within the selected window

bioRxiv preprint doi: https://doi.org/10.64898/2026.07.08.737286; this version posted July 9, 2026. The copyright holder for this preprint (which was not certified by peer review) is the author/funder, who has granted bioRxiv a license to display the preprint in perpetuity. It is made available under a CC-BY 4.0 International license.

Root Mean Square Acc	Square root of the mean of the squares of the acceleration intensity within the selected window
AUC	Area under the curve of the acceleration intensity series within the selected window, defined as the definite integral of the series, measuring velocity
Jerk	First time derivative of the acceleration intensity within the selected window
Time below Threshold	Proportion of time below an acceleration intensity threshold within the selected window, measuring immobility or underactive movement states. Threshold defined by grid searching.
Acc Range	Range of the acceleration intensity within the selected window
MSP	Mean power of the spectrum of the acceleration intensity series within the selected window
MSP 0.7-1.4	Mean power of 0.7-1.4 Hz frequency band of the spectrum of the acceleration intensity series within the selected window
MSP 1.4-2.8	Mean power of 1.4-2.8 Hz frequency band of the spectrum of the acceleration intensity series within the selected window
MSP 2.8-3.5	Mean power of 2.8-3.5 Hz frequency band of the spectrum of the acceleration intensity series within the selected window
Spectrum Entropy	Entropy of the spectrum of the acceleration intensity series within the selected window
Spectrum Variance	Variance of the spectrum of the acceleration intensity series within the selected window
Spectrum Flatness	Flatness of the spectrum of the acceleration intensity series within the selected window. Flatness is defined by ratio of the geometric mean to the arithmetic mean of the power spectrum. Values near 1 indicate a flat, noise-like spectrum; values near 0 indicate a peaky/tonal spectrum
Spectrum Smoothness	Smoothness of the spectrum of the acceleration intensity series within the selected window. Smoothness is defined by the mean of squares of the first-order difference of the power spectrum and measures the volatility of the spectrum

bioRxiv preprint doi: https://doi.org/10.64898/2026.07.08.737286; this version posted July 9, 2026. The copyright holder for this preprint (which was not certified by peer review) is the author/funder, who has granted bioRxiv a license to display the preprint in perpetuity. It is made available under a CC-BY 4.0 International license.

Spectrum Low Peak	Peak of the low frequency part (half of whole frequency band) of the spectrum of the acceleration intensity series within the selected window
Spectrum High Peak	Peak of the high frequency part (half of whole frequency band) of the spectrum of the acceleration intensity series within the selected window
Dominant Frequency Magnitude	The amplitude value corresponding to the frequency component with the strongest power in the spectrum of the acceleration intensity series within the selected window
Dominant Frequency Ratio	The ratio of the dominant frequency energy to the total spectrum energy of the acceleration intensity series within the selected window
Dominant Frequency Flatness	The flatness of the local frequency band (5 Hz long) centered by the dominant frequency of spectrum of the acceleration intensity series within the selected window
Dominant Frequency Entropy	The entropy of the local frequency band (5 Hz long) centered by the dominant frequency of spectrum of the acceleration intensity series within the selected window

Acceleration intensity is defined by the Euclidean norm of the recordings of all channels of the triaxial accelerometer

bioRxiv preprint doi: https://doi.org/10.64898/2026.07.08.737286; this version posted July 9, 2026. The copyright holder for this preprint (which was not certified by peer review) is the author/funder, who has granted bioRxiv a license to display the preprint in perpetuity. It is made available under a CC-BY 4.0 International license.

Supplementary Table 3: Parameters for Regression Models

Model	MATLAB Function	Parameters
Random Forest	TreeBagger	NumTrees', 200<br>'Method', 'regression'<br>'NumPredictorsToSample', Number of predictors/3<br>'MinLeafSize',5<br>'SampleWithReplacement', 'on'<br>'OOBPredictorImportance', 'on'
SVM	fitrsvm	KernelFunction', 'rbf'<br>'KernelScale', 'auto'<br>'BoxConstraint', 1<br>'Epsilon', 0.1<br>'Standardize', 'false'<br>'Solver', 'SMO'
Elastic Net	lasso	Alpha', 0.5<br>'CV', 10<br>'Lambda' , selected via 10-fold cross-validation (CV = 10) to minimize the mean squared error.<br>'Standardize', 'true'<br>'RelTol', 1e4<br>'NumLambda', 100<br>'Maxlter', 1e4
Fully Connected Network	trainNetwork	Network Structure: featureInputLayer(D)-fullyConnectedLayer(64)-reluLayer-fullyConnectedLayer(32)-reluLayer-fullyConnectedLayer(1)-regressionLayer<br>Loss function: Mean Square Error<br>'Optimizer', 'adam'<br>'MaxEpochs', '50'<br>'MiniBatchSize', 32

All models were implemented in MATLAB 2024b. D in featureInputLayer(D) refers to the dimension of input features.

bioRxiv preprint doi: https://doi.org/10.64898/2026.07.08.737286; this version posted July 9, 2026. The copyright holder for this preprint (which was not certified by peer review) is the author/funder, who has granted bioRxiv a license to display the preprint in perpetuity. It is made available under a CC-BY 4.0 International license.

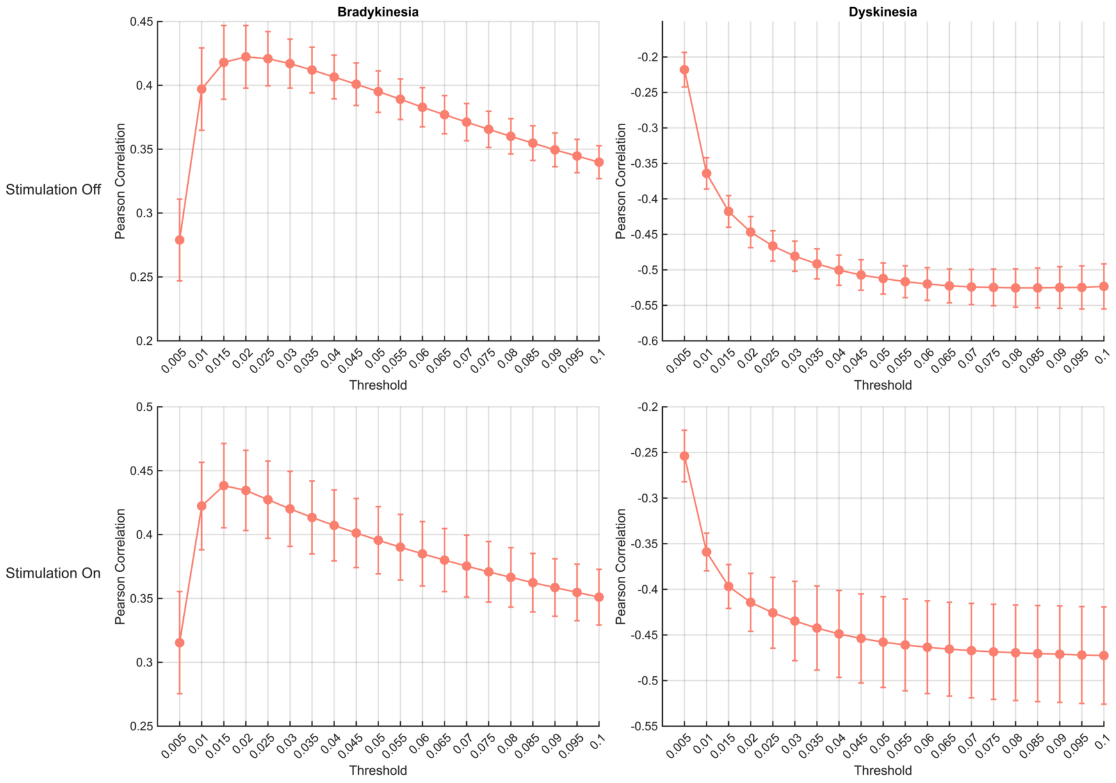

Supplementary Figure 1: Grid search optimization for the time below threshold accelerometer feature. Threshold range, 0.005 to 0.1; step, 0.005. The Pearson correlation coefficient between time below threshold and wearable bradykinesia or dyskinesia scores is used for optimization. The threshold with the highest absolute coefficient was selected as the optimal threshold. For bradykinesia, optimal thresholds were 0.02 (stimulation off) and 0.015 (stimulation on). Similarly for dyskinesia, optimal thresholds were 0.08 (stimulation off) and 0.1 (stimulation on).

bioRxiv preprint doi: https://doi.org/10.64898/2026.07.08.737286; this version posted July 9, 2026. The copyright holder for this preprint (which was not certified by peer review) is the author/funder, who has granted bioRxiv a license to display the preprint in perpetuity. It is made available under a CC-BY 4.0 International license.

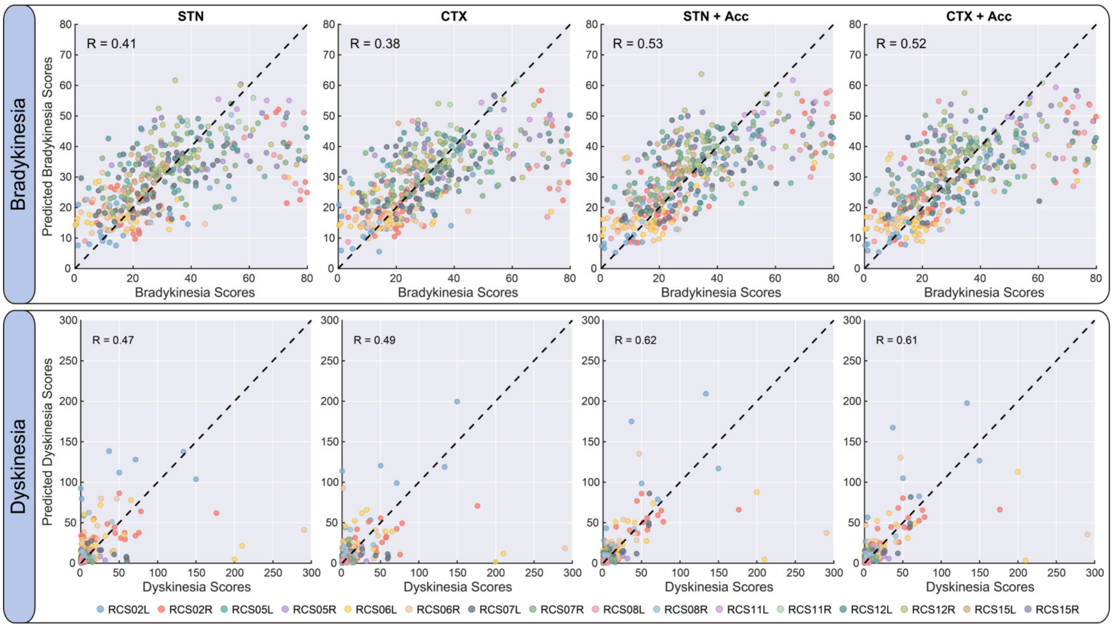

Supplementary Figure 2: Performance of the random forest regressor at predicting PKG derived bradykinesia and dyskinesia severity from device accelerometer and neural activity recordings. Predicted vs. true bradykinesia and dyskinesia scores for each hemisphere (different colours indicate different hemispheres), shown separately for predictions using STN neural features, cortical neural features and the combination of each of these features with device accelerometer features. For visualisation purposes the plotted dots are downsampled from the validation set of each fold of five-fold cross-validation, in a ratio of 1:50. The coefficient of determination was computed for all data points. Points on the diagonal (black dotted line) indicate perfect predictions.

bioRxiv preprint doi: https://doi.org/10.64898/2026.07.08.737286; this version posted July 9, 2026. The copyright holder for this preprint (which was not certified by peer review) is the author/funder, who has granted bioRxiv a license to display the preprint in perpetuity. It is made available under a CC-BY 4.0 International license.

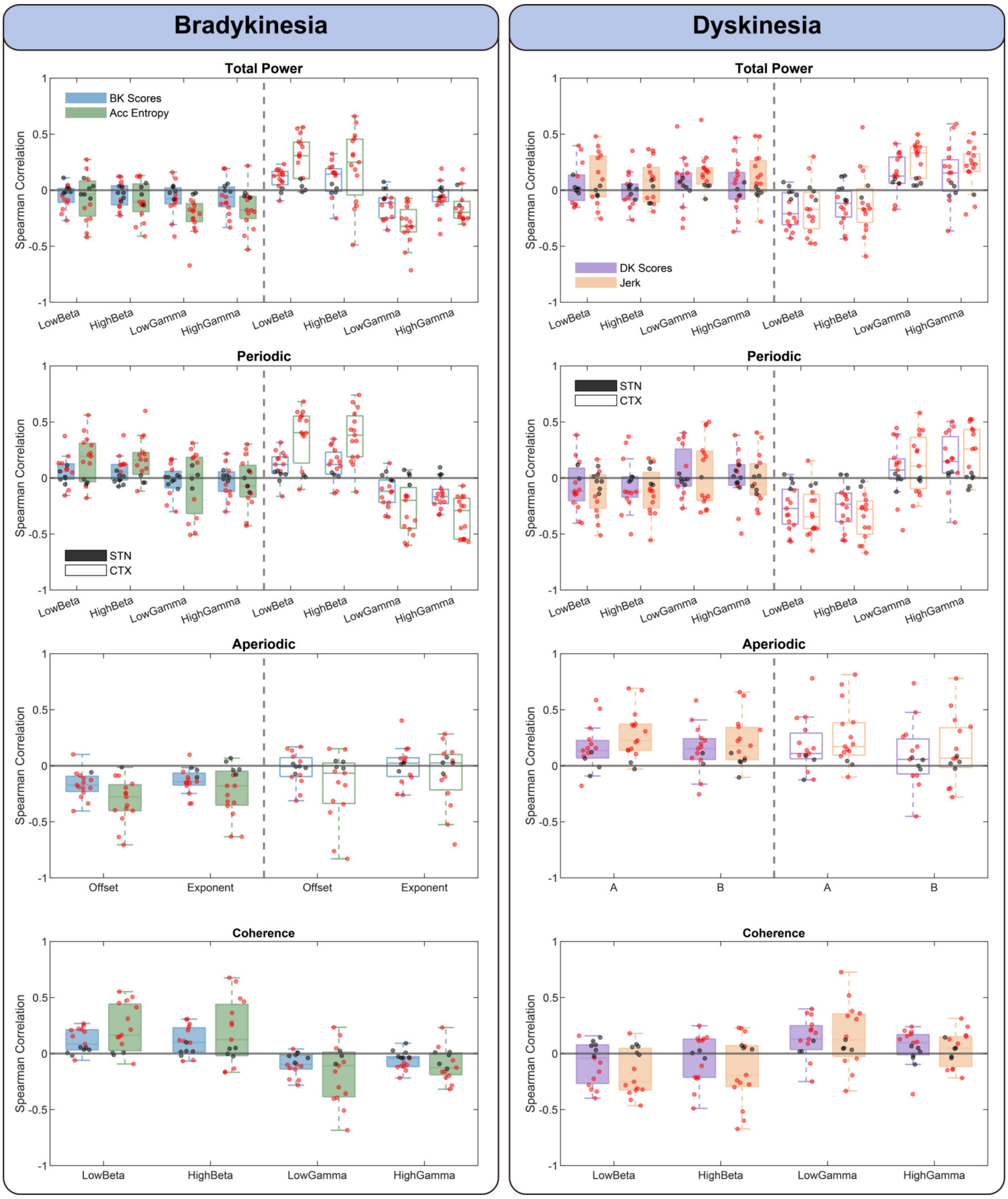

Supplementary Figure 3: Optimal accelerometer features recapitulate neural correlates of motor symptom severity during continuous STN DBS. Hemisphere-wise Spearman correlations between neural features and either PKG-derived motor scores or the corresponding optimal accelerometry-derived surrogate. Colours within each subplot denote correlation type (PKG score vs. device accelerometry; see legend). Dark shaded panels indicate STN features, whereas light shaded panels indicate motor cortical (CTX) features. Red points indicate hemispheres with statistically significant correlations after Benjamini–Hochberg false discovery rate correction ($q < 0.05$), whereas black points indicate non-significant associations. Offset, aperiodic offset; exponent, aperiodic exponent. STN, subthalamic nucleus; CTX, motor cortex.

bioRxiv preprint doi: https://doi.org/10.64898/2026.07.08.737286; this version posted July 9, 2026. The copyright holder for this preprint (which was not certified by peer review) is the author/funder, who has granted bioRxiv a license to display the preprint in perpetuity. It is made available under a CC-BY 4.0 International license.

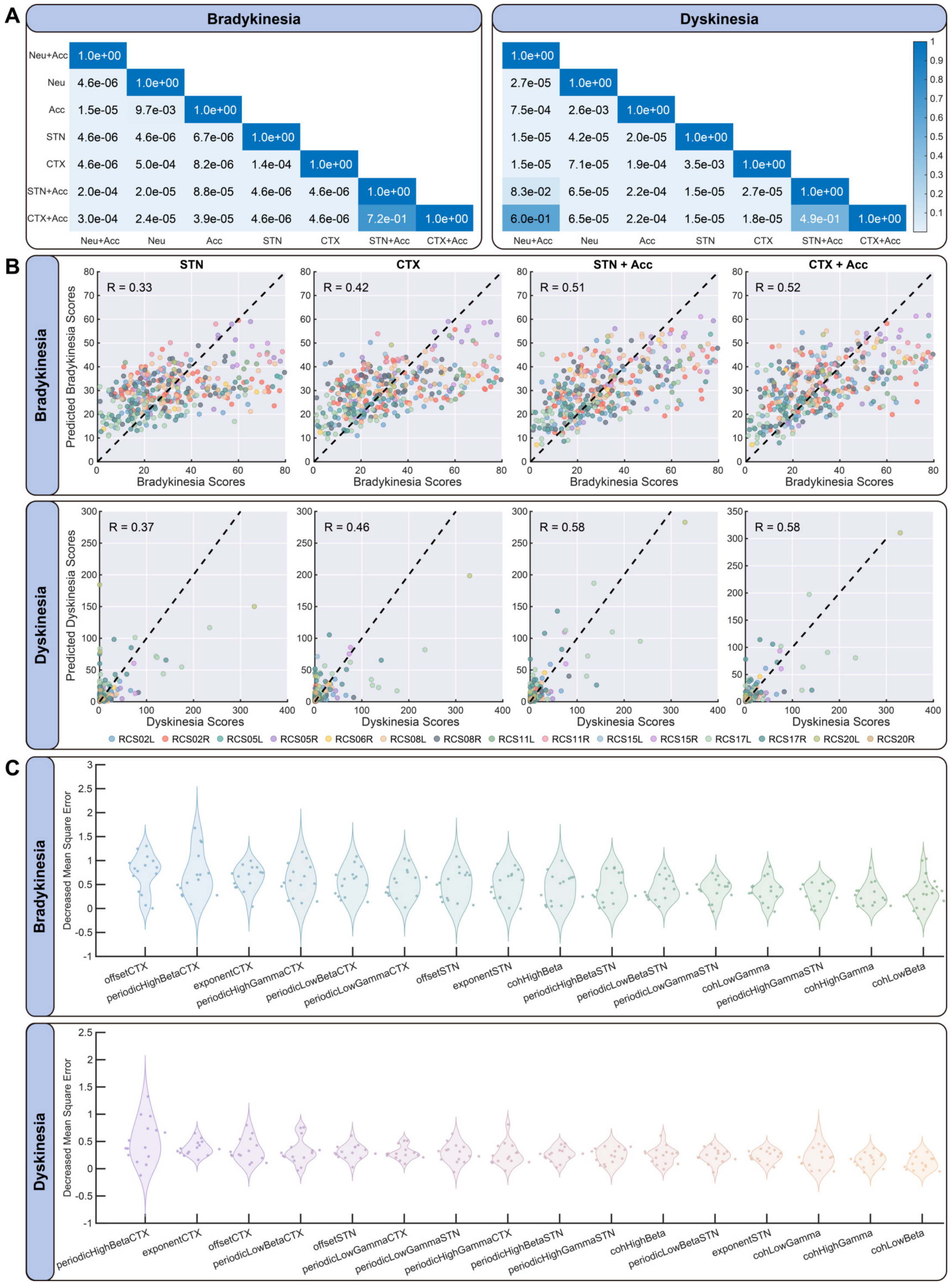

Supplementary Figure 4: **Device accelerometry outperforms neural activity features for predicting motor symptom severity during continuous STN DBS.** Data are shown for the Random Forest regressor. **A:** Pairwise comparisons of feature sets for bradykinesia and dyskinesia prediction. P-values were computed using Wilcoxon signed-rank tests on mean R^2 values across cross-validation folds. Multiple comparisons were corrected using the Benjamini–Hochberg false discovery rate, and q-values are reported. Colour indicates q-value. **B:** Predicted versus observed bradykinesia and dyskinesia scores across hemispheres for

bioRxiv preprint doi: https://doi.org/10.64898/2026.07.08.737286; this version posted July 9, 2026. The copyright holder for this preprint (which was not certified by peer review) is the author/funder, who has granted bioRxiv a license to display the preprint in perpetuity. It is made available under a CC-BY 4.0 International license.

models trained using STN neural features, cortical neural features and the combination of each of these features with device accelerometer features. For visualisation, a 1-in-50 random subsample of validation data is shown; R^2 values were computed using all observations. **C:** Feature importance for bradykinesia and dyskinesia prediction. Neural features are ordered by mean importance across hemispheres, quantified using out-of-bag (OOB) permutation importance. Each point represents one hemisphere.

bioRxiv preprint doi: https://doi.org/10.64898/2026.07.08.737286; this version posted July 9, 2026. The copyright holder for this preprint (which was not certified by peer review) is the author/funder, who has granted bioRxiv a license to display the preprint in perpetuity. It is made available under a CC-BY 4.0 International license.
